# Adherence to antiretroviral therapy and its associated factors among children at South Wollo Zone Hospitals, Northeast Ethiopia: a cross-sectional study

**DOI:** 10.1186/1471-2458-14-365

**Published:** 2014-04-15

**Authors:** Getachew Arage, Gizachew Assefa Tessema, Hiwot Kassa

**Affiliations:** 1 Department of Nursing, College of Health Sciences, Debre Tabor University, Debre Tabor, Ethiopia; 2 Department of Reproductive Health, Institute of Public Health, University of Gondar, Gondar, Ethiopia; 3 Department of Nursing, College of Medicine and Health Sciences, University of Gondar, Gondar, Ethiopia

**Keywords:** HIV, Adherence, ART, Children, Ethiopia

## Abstract

**Background:**

Poor adherence to antiretroviral therapy negatively affects the suppression of viral replication. It increases risks of drug resistance, treatment failure, Acquired Immuno Deficiency Syndrome (AIDS)-related morbidity and mortality among children. This study assessed the level of adherence to antiretroviral therapy and its associated factors among children at hospitals in South Wollo Zone, Northeast Ethiopia.

**Methods:**

An institution-based cross-sectional study was conducted among Human Immunodeficiency Virus (HIV)-infected children in April 2013. A total of 464 children who were taking Antiretroviral Therapy (ART) in the hospitals were included. Data were collected using pretested and structured questionnaires using a face-to-face interview method. Descriptive and summary statistics were employed. Bivariate and multiple logistic regressions were computed. Odds ratios and their 95% confidence intervals were computed to determine the level of significance.

**Results:**

Of the 464 study samples, 440 children with their caregivers were included in the final analysis. A total of 78.6% of the caregivers reported that their children were adherent to antiretroviral therapy in the month prior to the interview. Caregivers' knowledge about antiretroviral treatment [AOR = 2.72(95% CI: 1.82, 5.39)], no current substance use of the caregivers [Adjusted Odds Ratio (AOR) = 2.21(95% Confidence Interval (CI): 1.34, 7.13)], proximity to the health care facility [AOR = 2.31(95% CI: 1.94, 4.63)], if the child knows HIV-positive status [AOR = 3.47(95% CI: 2.10, 6.81)] and caregiver’s educational status [AOR = 0.59(95% CI: 0.21, 0.82)] were significantly and independently associated with adherence of children to antiretroviral therapy.

**Conclusion:**

Adherence of antiretroviral therapy in this study was comparable to other studies conducted in developing countries. Caregiver’s knowledge about antiretroviral therapy, no current use of substances, close proximity to health facilities, and letting child’s know his/her HIV status improves adherence to antiretroviral therapy. Health care providers should educate caregivers about antiretroviral therapy and encourage HIV positive status disclosure to the child.

## Background

Globally, of the estimated 34 million people living with HIV in 2011, 3.3 million were children under the age of 15 years. More than 90cph of these children were residing in sub-Saharan Africa [1]. In 2010, Ethiopia had 1,216,908 people living with HIV of which 79,875 (6.5%) were children [2]. Antiretroviral Therapy (ART) improves the prognosis of HIV-infected individuals, reduces HIV-related morbidity and mortality, and reduces other opportunistic infections [3-5].

Inadequate adherence increases the risk of drug resistance and treatment failure [6]. Therefore, optimum adherence is essential for sustainable success of ART [7]. Suboptimal adherence among children is common in both developed and developing countries [8]. Taking ≥ 95% of prescribed doses is recommended for optimal virologic suppression and minimizes the rate of treatment failure [6,9] and decreases risk of virologic failure by more than 50% [10].

Adherence is a serious challenge for those receiving ART especially children. Factors associated with paediatric ART adherence can be related to caregivers, children themselves, the medication/regimen, socioeconomic, or service delivery issues [11-14].

Unlike adults, young children rely upon their caregivers for their medicines. However, these caregivers may also be ill [14]. In addition, children may not be able to take pills and syrup formulations because of the large size of tablets and poor palatability of syrups [15,16]. Furthermore, parents may have a poor understanding of the need to take the medication and they may be unwilling to disclose the child’s HIV-positive status to the child or others involved in the care. This may create problems in administering doses while the parent is at work or the child at school (9). HIV develops resistance if the concentration of drugs in the blood is low. Hence, optimal adherence to ART is essential to ensure high level of drugs by taking medication correctly and consistently (10).

As a result, the guideline for paediatric HIV care and treatment in Ethiopia recommends at least two adherence sessions which include caregiver education and counseling before ART initiation. The same guideline also recommends adherence reinforcement at each follow-up visit. Moreover, the limited availability of second-line therapy in resource-limited settings like Ethiopia emphasizes the importance of adherence and preservation of first-line regimens. Therefore, assessment and support of adherence is fundamental to successful antiretroviral therapy and prevention of drug resistance [6]. The findings of this study are particularly helpful to those clinicians who work with paediatric HIV positive patients in third world countries. Hence, this study assessed the level of adherence to ART and associated factors among children at hospitals in South Wollo Zone, Northeast Ethiopia.

## Methods

A facility-based cross-sectional study was conducted in April 2013 at paediatric ART clinics in the three hospitals (Dessie Referral Hospital, Borumeda District Hospital, and Hidar 11 District Hospital) that provide ART services in South Wollo Zone, Northeast Ethiopia. These three hospitals provide services to about 15,000, 1,600 and 2,500 clients living with HIV registered at chronic care follow-up clinics (treated and untreated), respectively. There were 1210 children on chronic HIV care and support follow up at the three hospitals (900 at Dessie, 130 at Borumeda, and 180 at Hidar-11 hospitals). Of these 1210 children, 464 (324 at Dessie, 58 at Borumeda, and 82 at Hidar-11 hospitals) were on ART. Each hospital has its own case managers, adherence supporters, support groups for mothers, and data clerks. Children aged 2 months to 14 years and who were receiving ART for at least one month were included in the study.

The sample size was calculated by considering the assumptions for single population proportion formula: the proportion (P) =80.9% of adherence rate in Northwest Ethiopia [17], Z = standard normal distribution value at 95% confidence level of Za/2 = 1.96, 5% of absolute precision, and 10% non-response rate. Hence, the total sample size was 263. However, there were 464 children who were taking ART in these hospitals and therefore, all were included.

Pretested and structured questionnaires using face-to-face interviewing with caregivers were used for data collection. The questionnaire was partly adapted from the AIDS Clinical Trial Group (ACTG) adherence follow-up questionnaire [18] and review of similar literature. Pre-testing of the questionnaire was undertaken on 24 caregivers at the Dessie Health Center before the actual data collection. Medical records were reviewed to collect clinical data such as WHO clinical stage and the CD4 count of children. Adherence was measured by the caregivers’ report of a one-month recall of missed doses prior to the date of the interview.

Data were collected by five diploma nurses (supervised by three B.Sc nurses). A two days comprehensive training was given to data collectors and supervisors. The questionnaire was first prepared in English and then translated into Amharic (the local language), and back into English to ensure consistency.

The questionnaires were coded and entered into EPI Info version 3.5.3 statistical software and then exported to SPSS windows version 16 for further analysis. Data were summarized and presented using descriptive statistics. Bivariate and multiple logistic regressions were computed to identify the presence and strength of associations. Odds ratios with 95% CI were computed and variables having p-values less than 0.05 in the multiple logistic regression models were considered significantly associated with the dependent variable (adherence).

### Operational definitions

*Adherence* to ART in this study was when the children took ≥95% of the prescribed doses in the month prior to the interview. *Non- adherence to ART was* when the child took less than 95% of the total doses or missed more than 3 doses in the month prior to the interview. In addition, if a child missed a single dose in the past three and seven days, it was considered non-adherent.

Knowledge about ART was categorised by asking eight questions. Those respondents who scored greater than or equal to the mean for the knowledge questions were considered as *knowledgeable,* otherwise *not.*

Attitude about ART was categorised based on five questions using a Likert scale method and the mean scores were computed and then dichotomized into favourable (score ≥ mean value) or unfavourable (score < mean value).

### Ethical considerations

Ethical clearance was obtained from the Ethical Review Committee of the College of Medicine and Health Sciences, University of Gondar. An official letter of cooperation was granted to the administrative offices of the three hospitals. Verbal informed consent was obtained from each participant before the start of the interview. Due to the high illiteracy, it was considered impractical to obtain written consent from each study participant. Also, assent was secured from those children older than seven years and were aware of their sero-postive status. All interviews were conducted in a private room and confidentiality was insured.

## Results

### Socio-demographic and clinical characteristics of the study participants

Of the 464 study participants, 440 children along with their caregivers were included in the analysis, yielding a response rate of 94.8%. With the twenty-four excluded cases, eighteen caregivers did not know the dosing history for the past month and six questionnaires were discarded for being incomplete. Two hundred eighty one (63.9%) of the caregivers were females. The mean age of the children was 9.4 years (SD = 5.13) and 54.8% of them were between the ages of 10–14 years. Half (50.7%) of the caregivers were unable to read and write. Two hundred and fourteen (48.6%) of the children were at WHO clinical stage II. One hundred fifty-nine (36.1%) children had a CD4 count of <200 cells/mm3 at ART initiation and 56% had a current CD4 count of ≥500 cells/mm^3^ (Table 1).

**Table 1:** Socio-demographic characteristics of the study participants at South Wollo Zone hospitals, Northeast Ethiopia, 2013 (n = 440)

**Variables**	**Frequency**	**Percentage**
**Sex of caregiver**		
Male	159	36.1
Female	281	63.9
**Age of caregiver (in years)**		
<20	16	3.6
21-30	118	26.8
31-40	162	36.8
≥41	143	32.6
**Ethnicity**		
Amhara	409	93.0
Tigire	18	4.1
Oromo	10	2.3
Others*	3	0.7
**Marital status**		
Single	60	13.6
Married	240	54.5
Divorced	70	15.9
Widowed	70	15.9
**Religion**		
Muslim	245	55.7
Orthodox Christian	182	41.4
Protestant	13	3.0
**Educational status of the caregiver**		
Can’t read and write	223	50.7
Primary school(1–8 grade)	116	26.4
Secondary or above	101	23.0
**Substance use of caregivers**		
User	44	10.0
Non-user	396	90.0
**Caregivers income (Ethiopian Birr)**		
<=300	46	10.5
300-999	237	53.9
≥1000	157	35.7
**Occupation**		
Merchant	106	24.1
House wife	90	20.5
Farmer	165	37.5
Government employee	65	14.8
Other**	14	3.2
**Age of children (in years)**		
<4	34	7.7
5-9	156	37.5
10-14	241	54.8
**Sex of children**		
Male	210	47.7
Female	230	52.3
**Children’s caregiver**		
Biological parents	343	78.0
Other relatives and guardian	97	22.1
**WHO Clinical stage**		
Stage I	60	13.6
Stage II	214	48.6
Stage III	161	36.6
Stage IV	5	1.1
**Baseline CD4 count**		
<200	159	36.1
200-499	224	50.9
≥500	57	13.0
**Current CD4 count**		
<200	6	1.4
200-499	187	42.5
≥500	247	56.1

*Gurage, Afar **Students, daily laborers.

### Knowledge and attitude about ART treatment

Two-thirds (67%) of the participants knew about ART before their child was diagnosed. The majority (90%) of the respondents were knowledgeable and had a favourable attitude towards ART. Three hundred sixty-two (82.3%) participants reported that taking ART incorrectly would result in resistance to the drugs.

### Adherence to ART among children

Based on the caregivers’ report, a total of 78.6% (95% CI: 74.8%, 82.4%) children were reported to have an adherence rate of ≥95% in the month prior to interview. The adherence rates to ART among children in the past three and seven days of the interview date were 95.9% and 89.8%, respectively.

### Reasons for non-adherence

**Figure 1:**
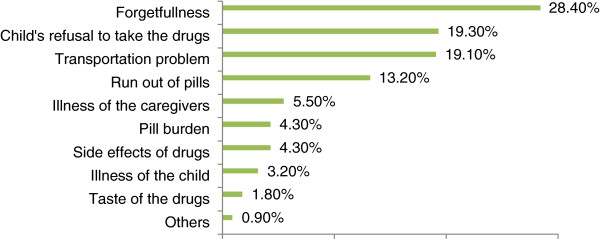
Reasons for non-adherence to ART among children at South Wollo Zone Hospitals, Northeast Ethiopia, 2013.

The commonly mentioned reasons for missing these medications were: forgetfulness (28.4%), child’s refusal to take the drugs (19.3%); and lack of transportation access to the facilities (19.1%) (Figure 1).

### Factors associated with adherence to ART among children

In multiple logistic regression analysis, current substance use of the caregivers, disclosure of HIV status to the child, knowledge of caregivers about ART treatment, distance from the health facility, and educational status of secondary schooling or above of the caregivers, and current CD4 count of 500 or more were significantly and independently associated with adherence.

Children whose caregivers were not currently using substances were 2.2 times more likely to be adherent to ART than those whose caregivers used substances like alcohol, chat and/or cigarettes [AOR = 2.21(95% CI: 1.94, 5.39)]. Children who knew their HIV status (disclosed) were 3.6 times more likely to be adherent than their counterparts [AOR = 3.47(95% CI: 2.10, 6.81)].

The present study also showed that children who came from a distance of less than 10 km were 2.3 times more likely to be adherent to ART compared to those who came from 10 km or above [AOR = 2.31(95% CI: 1.94, 4.63)].

If the caregiver was knowledgeable about ART treatment, the child was 2.7 times more likely to be adherent to ART [AOR = 2.72(95% CI: 1.82, 7.13)]. Those children whose caregivers had secondary or above educational status were 0.41% less likely to be adherent than those children with caregivers who did not read and write [AOR = 0.59(95% CI: 0.21, 0.83)]. Those children whose current CD4 count of 500 or greater were 1.89 times more likely to be adherent than those children with CD4 count less than 500 [AOR = 1.89(95% CI:1.34, 3.87)] (Table 2).

**Table 2:** Crude and adjusted odds ratios (OR) and 95% confidence intervals (CI) of determinants adherence to HAART at South Wollo Zone hospitals, Northeast Ethiopia, 2013 (n = 440)

**Variables**	**Adherence status**		**OR (95% CI)**			
	**Adherent**	**Non- adherent**	**COR(95% CI)**	**P value**	**AOR(95% CI)**	**P-value**
**Sex of the caregivers**				0.145		
Male	119	40	1			
Female	227	54	1.4(0.88, 2.25)			
**Age of the caregivers**				0.16		
<30	106	28	1			
31-40	133	29	1.29(0.73, 4.07)			
≥41	102	33	0.49(0.31, 2.51)			
**Educational status of the caregivers**				0.022		0.031
Can’t read and write	175	48	1		1	
Elementary school	97	19	1.4(0.77,2.52)		1.23(0.32, 2.66)	
Secondary or above	74	27	0.75(0.43,1.29)		0.59(0.21,0.83)*	
Substance use of the caregivers*				0.033		0.008
Used	29	15	1		1	
Not used	317	79	2.07(1.07,4.05)		2.21(1.34,5.39)*	
**Caregivers knowledge about ART**				0.004		0.023
Knowledgeable	320	77	2.71(1.40, 5.25)		2.72(1.82, 7.13)*	
Non knowledgeable	26	17	1		1	
**Attitude of caregivers towards ART**				0.030		
Favourable	294	88	0.38( 0.28, 6.24)			
Unfavourable	52	6	1			
**Taking medication besides ART**						
Yes	26	4	1			
No	320	90	0.54(0.52,5.37)			
**Distance from the health facility**				0.001		0.003
<10Km	246	45	2.6(1.68,4.22)		2.31(1.94, 4. 63)*	
≥10Km	100	49	1		1	
**Disclosure of HIV status**				0.041		0.029
Yes	216	28	3.9(2.39,6.4)		3.47(2.10, 6.81)*	
No	130	66	1		1	
**CD4 count at the start of treatment**				0.18		
<200	118	41	1			
200-499	179	45	1.3(0.85, 2.25)			
≥500	49	8	2.12(0.93,4.87)			
**Getting support**						
Yes	73	11	2.01(1.02,3.98)			
No	273	83	1			
**Current CD4 count**				0.03		0.022
<500	139	54	1		1	
≥500	207	40	2.01(1.27, 3.19)		1.89(1.34, 3.87)*	

*p value < 0.05.

## Discussion

This study assessed the rate of antiretroviral therapy adherence and its associated factors among children at South Wollo Zone Hospitals, Northeast Ethiopia. The level of adherence to ART treatment was found to be 78.6% in the past one month caregivers’ reports. This finding is comparable to similar studies conducted in Ethiopia (80.9%) [17], Nigeria (80%) [19], Togo (80%) [20], and Vietnam (75.1%) [21].

This finding is also comparable with a systematic review of paediatric ART adherence studies in middle- and low-income countries (75%) [22]. Nevertheless, it was lower than studies conducted in the United States (84%) [22], Jamaica (87.5%) [23], New Delhi (91.4%) [24] but higher than studies conducted in Brazil (50.5%) [25], and Kenya (44.2%) [26].

In the present study, caregivers’ forgetfulness was reported as the major reason (28.4%) for missing doses. Studies conducted in Brazil, Kenya, and Nigeria [25-27] supported this finding. This could be due to the caregivers being busy with their daily routines which, in turn, lead the caregivers to easily forget to administer the pills as prescribed. Similar to other studies [28-30], child refusal to take medication (19.3%) and lack of transport access to reach facilities (19%) lead them to miss doses.

A number of factors were reported as predictors of adherence among children on ART. In the present study, if the caregiver uses substances then the child was less likely to be adherent to ART. A studies conducted in Vietnam [21], Nepal [30], and South Africa [31] showed similar findings. This might be due to substance use could lead them to forget administration of the drugs. Forgetfulness is one of the reasons for missing doses in children as shown by this study (28.4%).

The findings also showed that those children who have been disclosed of their HIV status were about four times more likely to be adherent to ART than those who had not been disclosed of their HIV status. This finding is supported in studies conducted at Uganda [32] and Democratic Republic of Congo [16]. This may be seen from two overlapping, yet separate, angles. Firstly, children who knew they are HIV-positive status can be more concerned on their health and could aware that these drugs are helpful. Secondly, nondisclosed children might not understand the rationale behind taking drugs and become resistant to take them as they fail to understand why they take medicine while feeling apparently healthy.

This study also showed that children’s adherence to ART is associated with distance from the health facilities which is in line with findings from Ethiopia [28], Nepal [30], and South Africa [31]. This could happen because the clients miss appointments due to difficulties in reaching the treatment centres. Lack of transportation, long traveling distance, and geographical inaccessibilities might be the reasons.

In the present study, caregivers knowledge about ART treatment was associated with the child’s adherence to ART. This finding is consistent with that of a study conducted in Addis Ababa [28]. If caregivers do not understand benefits of ART to children, they might be poor motivated to administer drugs timely and regularly.

Unlike other studies [30,33], in this study educational status of caregiver was negatively associated with adherence to ART. Children whose caregivers had secondary and above education were less likely to adhere to ART compared to children whose caregivers were not able to read and write. This could be explained by the idea that these caregivers were more likely employed and thus spend less time with the child to administer the drugs consistently or forget doing so amidst busy days.

In the present study, children whose current CD4 count of 500 or greater were about two times more likely to be adherent than those children with CD4 count less than 500. It could be surmised that long-term adherence leads to higher CD4 counts i.e. CD4 increases as a result of optimal adherence to ART.

However, the present study does have some inherent limitations. Due to financial constraints, adherence was assessed through a self-reporting adherence questionnaire instead of other more objective tools. Caregivers’ reports might lead to overestimated adherence. Though efforts made during data collection to minimize the social desirability and recall biases, these may not be eliminated. This study did not assess the adherence related to the correct timing for ART drug administration. The cross-sectional nature of the study which used a snapshot of adherence at one point in time may hinder the accuracy of adherence.

## Conclusion

Adherence to ART in the study area was comparable to other studies conducted in developing countries. The caregiver’s knowledge about ART, non-usage of substances, proximity to health facilities, and disclosing the child’s HIV status were associated with adherence to ART. Health care providers should educate caregivers about ART and avoidance of substance use. Further attempts are also needed to encourage caregivers to disclose the HIV status to the children.

## Competing interests

The authors declare that they have no competing interests.

## Authors’ contributions

GA wrote the proposal, participated in data collection, analyzed the data and drafted the paper. GAT and HK approved the proposal with revisions, participated in data analysis and revised subsequent drafts of the paper. All authors read and approved the final manuscript.

## Pre-publication history

The pre-publication history for this paper can be accessed here:

http://www.biomedcentral.com/1471-2458/14/365/prepub
